# Hepatitis virus infection and age-related cataract

**DOI:** 10.1038/s41598-017-13283-6

**Published:** 2017-10-12

**Authors:** Sangshin Park, Nam-Kyong Choi

**Affiliations:** 1 Center for International Health Research, Rhode Island Hospital, The Warren Alpert Medical School of Brown University, Providence, RI 02903 USA; 2 Department of Pediatrics, The Warren Alpert Medical School of Brown University, Providence, RI 02903 USA; 3 Department of Health Convergence, Ewha Womans University, Seoul, Republic of Korea

## Abstract

This study was performed to investigate the relationships of hepatitis B (HBV) and hepatitis C virus (HCV) infection to age-related cataract, and to assess whether liver damage mediates the hepatitis-cataract association. This study analyzed data in the Korea National Health and Nutrition Examination Survey 2010–2012 on 10,037 participants aged ≥40 years. We performed mediation analysis to address the contribution of serum markers of liver damage, high aspartate (AST, >49.9 IU/L) and alanine aminotransferase (ALT, >56.1 IU/L), to the relationships of HBV and HCV infection to cataract. Odds ratios (ORs) for nuclear and any cataract with HBV infection were 1.09 [95% confidence interval (95CI) = 1.02–1.16] and 1.07 (95CI = 1.00–1.14), respectively, compared to HBV uninfection; ORs with HCV infection were 1.35 (95CI = 1.18–1.55) and 1.40 (95CI = 1.12–1.76), respectively. High AST completely mediated the HBV infection-any cataract association. The significant relationships of HCV infection with nuclear and any cataract were formed only by their direct effects, not by mediation effects of high AST or ALT. HBV and HCV infection was significantly associated with nuclear and any cataract. High AST significantly mediates the effects of HBV infections on any cataract outcome, but the associations of HCV infection with nuclear and any cataract were not mediated by high AST or ALT.

## Introduction

Cataract is one of the well-known major causes of vision impairment and blindness. In 2010, approximately 33% of 32.4 million blindness cases and 18% of 191 million moderate and severe vision impairment cases worldwide were caused by cataract; more than 40% of blindness was caused by cataract in some regions, such as Southern and Southeastern Asia^1^. In the United States, cataract-related claims account for about 60% of eye care charges in Medicare population^2^. Age-related cataract has been and is consistently expected to be a major public health issue worldwide with the increase of the elderly population.

Liver cancer is the second leading cause of death from cancer worldwide, estimated at approximately 746,000 deaths (9% of total) in 2012^3^. Of 14 million new cancer cases in 2012 worldwide, 420,000 and 170,000 cases were attributable to HBV and HCV, respectively; 73% of liver cancer was caused by these hepatitis viruses^4^. Approximately 248 million people (3.6% of total) are estimated to be hepatitis B surface antigen (HBsAg) positive in the worldwide general population^5^. More than 185 million people (2.8% of total) are estimated to have antibody to HCV (anti-HCV) worldwide, and temporal trends between 1990 and 2005 indicate an increase in anti-HCV^6^. Therefore, HBV and HCV are also considered to be an important problem for worldwide public health in less developed countries in particular^3,5,6^.

HBV and HCV infection leads to hepatic inflammation, fibrosis, and some complications, such as end-stage liver disease and hepatocellular carcinoma^6–9^. Aspartate aminotransferase (AST) and alanine aminotransferase (ALT) are widely used to evaluate hepatitis activity^10^. Indeed, several epidemiologic studies identified a relationship between HBV and HCV infection and AST and ALT levels^7–9,11^. Considering that liver dysfunction is a risk factor for cataract formation^12^, we inferred that HBV and HCV infection has some relationship with cataract formation, and that liver damage is involved in this association of HBV or HCV and cataract. But little evidence suggests that HBV and HCV infection is associated with age-related cataract. The objectives of this study were to (1) assess the relationships between HBV and HCV and age-related cataract, and (2) examine whether high AST and ALT levels mediate the relationship between hepatitis virus infection and age-related cataract in a Korean population greater than or equal to 40 years old.

## Results

Of 10,037 participants examined for HBV, 386 (3.9%) were infected with HBV. Of 3,166, 41 (1.3%) were infected with HCV (Table 1). Age, house income, and AST and ALT levels were significantly different between participants infected and uninfected with HBV and HCV. Education status, diabetes mellitus, and obesity differed for participants infected and uninfected with HBV. Sun exposure and nuclear and any cataract were different for participants infected and uninfected with HCV. Only one participant was infected with HBV and HCV simultaneously.

**Table 1:** Characteristics of study participants.

	Hepatitis status					
	Hepatitis B virus		*P* value^b^	Hepatitis C virus		*P* value^b^
	Uninfected	Infected		Uninfected	Infected	
N (%)	9651 (96.2)	386 (3.9)		3125 (98.7)	41 (1.3)	
Women, %	56.1	50.0	0.019	56.7	51.2	0.48
Age, y	56.6 ± 10.8	54.5 ± 9.8	< 0.001	56.8 ± 10.8	61.0 ± 11.5	0.022
Smoking amount,^a^ pack-years	3.1 ± 4.8	3.2 ± 4.9	0.55	3.0 ± 4.4	2.8 ± 4.1	0.64
Alcohol consumption,^a^ g/d	2.5 ± 3.2	2.6 ± 3.5	0.57	2.4 ± 3.0	2.1 ± 2.3	0.61
Metabolic equivalence task,^a^ MET-hr/wk	594 ± 1604	593 ± 1623	0.57	504 ± 1380	344 ± 1108	0.47
Education						
Elementary school	31.5	25.5	0.028	30.5	41.0	0.06
Middle school	15.7	15.0		14.9	23.1	
≥High school	52.9	59.5		54.5	35.9	
Household income ≥$3000/mo, %	49.8	57.9	0.002	49.5	32.5	0.033
Diabetes mellitus, %	11.7	7.3	0.008	11.4	17.1	0.22
Obesity (BMI ≥25 kg/m^2^), %	34.7	40.3	0.024	34.8	43.9	0.23
Sun exposure						
<2 h/d, %	60.9	60.9	0.93	66.4	46.3	0.015
2–5 h/d, %	23.2	23.8		22.7	31.7	
>5 h/d, %	15.9	15.3		10.9	22.0	
Family history of eye diseases,^c^ %	19.9	17.9	0.32	21.3	12.2	0.16
Liver function						
AST,^a^ IU/L	21.7 ± 7.1	26.4 ± 12.2	<0.001	21.7 ± 7.4	27.7 ± 13.6	<0.001
High AST (AST >49.9 IU/L), %	2.5	9.8	<0.001	2.9	17.1	<0.001
ALT,^a^ IU/L	18.9 ± 9.1	23.9 ± 14.2	<0.001	18.8 ± 9.2	26.4 ± 17.1	<0.001
High ALT (ALT > 56.1 IU/L), %	2.8	9.1	<0.001	3.0	9.8	0.038
Cataract, %						
Cortical	9.3	7.8	0.32	10.7	4.9	0.31
Nuclear	26.6	28.0	0.55	29.0	63.4	<0.001
Mixed	6.6	4.2	0.06	7.6	7.3	1.00
Any	42.2	39.9	0.37	47.0	78.1	<0.001

Data are arithmetic or geometric^a^ mean ± SD or %. ^b^Continuous variables were analyzed by Wilcoxon rank-sum tests, and categorical variables were analyzed by Chi-square or Fisher’s exact tests. ^c^Glaucoma, cataract, strabismus, retinopathy, blepharoptosis, or other eye diseases.

**Figure 1:**
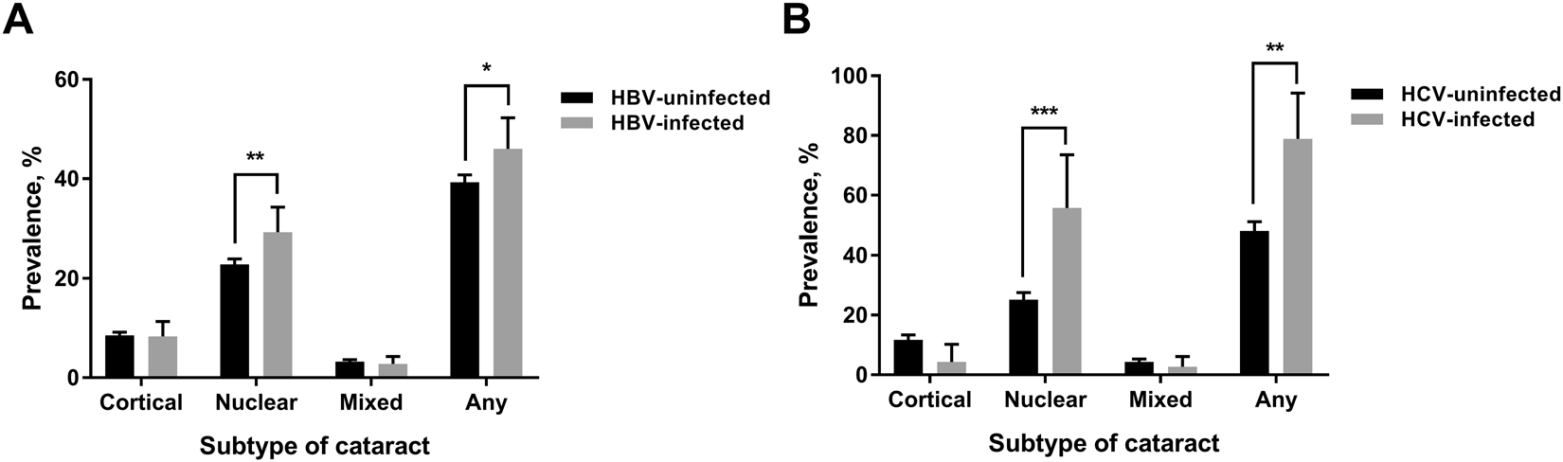
Adjusted prevalence of cataract subtype according to the status of (**A**) hepatitis B virus (HBV) infection and (**B**) hepatitis C virus (HCV) infection. The error bars represent 95% CIs. Adjusted for age, sex, smoking amount, alcohol consumption, metabolic equivalence task, education, household income, diabetes mellitus, obesity, sun exposure, and family history of eye diseases. *P value < 0.01; **P value < 0.001; ***P value < 0.001.

After adjusting for confounders, the prevalence of nuclear (uninfected, 23.2%; infected, 28.8%) and any (uninfected, 39.6%; infected, 44.9%) cataract was significantly higher in participants infected with HBV, compared to those uninfected [Fig. 1(A)]. The prevalence of nuclear (uninfected, 26.5%; infected, 58.1%) and any (uninfected, 47.3%; infected, 77.7%) cataract was also significantly different for participants infected and uninfected with HCV [Fig. 1(B)]. Therefore, we performed mediation analysis to examine the roles of high AST and ALT in the relationship between HBV and HCV and nuclear and any cataract.

**Figure 2:**
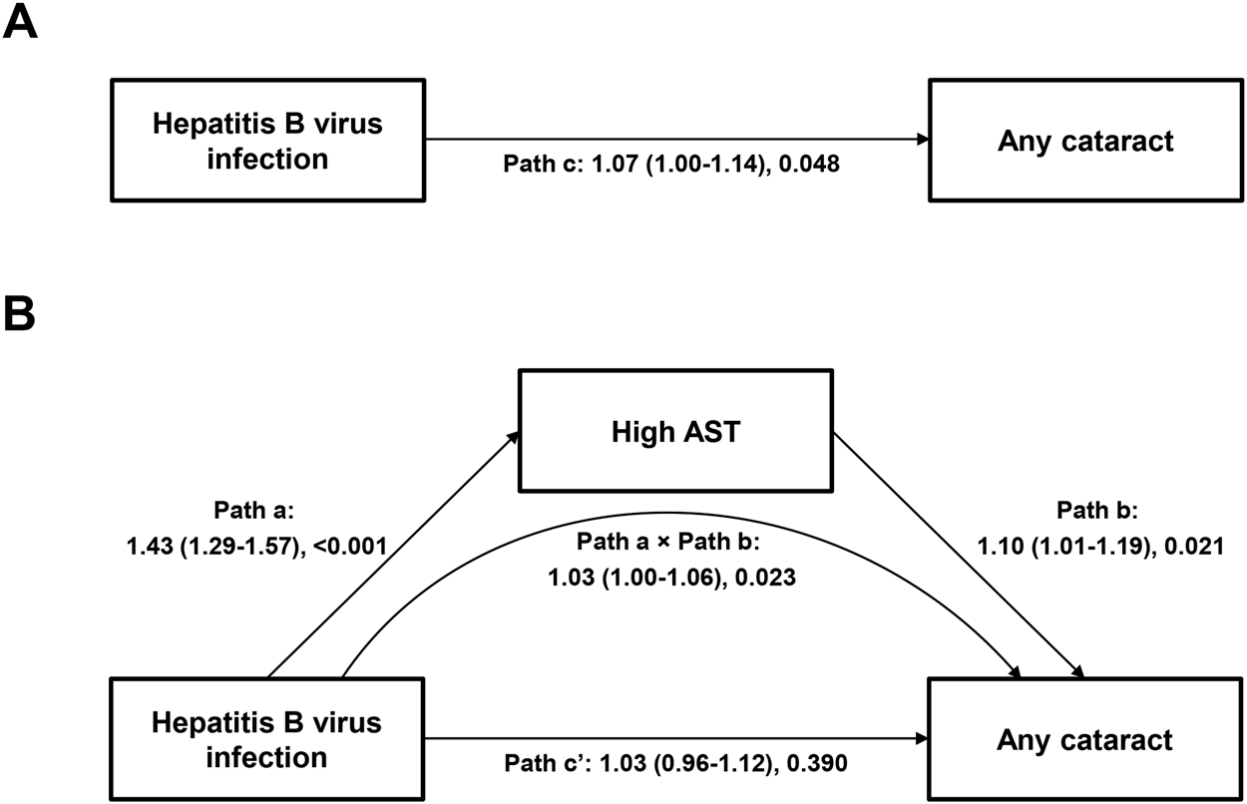
Mediation pathway in the association between hepatitis B virus (HBV) infection and any cataract through aspartate aminotransferase (AST). (**A**) Total effect (path c) of HBV infection on cataract, (**B**) Direct (path c’) and indirect (path a × path b) effects of HBV infection on any cataract through high AST (>49.9 IU/L). Path a indicates the direct effect of HBV infection on high AST. Path b indicates the direct effect of high AST on any cataract. Goodness of fit measures for all mediation models were: the model’s relative χ^2^ = 1.63 with *P* value = 0.133, RMSEA = 0.008, and CFI = 0.998. Direct, indirect, and total effects and percent mediated are also shown in Table 2.

In the mediation analysis, we found the significant total effects of HBV and HCV on nuclear [odds ratio (OR), 1.07 to 1.09] and any (OR, 1.35 to 1.40) cataract (Table 2; Fig. 2). High AST and ALT significantly and borderline significantly, respectively, mediated the relationship between HBV infection and any cataract: complete mediation effects were established for this relationship. However, no significant mediation effects were observed in the relationship between HBV and nuclear cataract. Regarding the HCV infection predictor, indirect effects were not significant. Goodness of fit measures for all mediation models were: the models’ relative χ^2^ = 1.34 to 1.97 with *P* values > 0.05, the root mean square error of approximation (RMSEA) = 0.006 to 0.010, and the comparative fit index (CFI) = 0.992 to 0.999. These indicate good approximate model fit.

**Table 2:** Estimated mediation of the association between hepatitis B virus (HBV) or hepatitis C virus (HCV) infection and cataract by liver function^a^.

Outcome: subtype of cataract	Total effect (through path c)^b^	Mediator	Direct effect (through path c’)^b^	Indirect effect (through path a and b)^b^
Predictor: HBV infection				
Nuclear	**1.09 (1.02–1.16), 0.008**	High AST	1.08 (1.00–1.16), 0.051	1.01 (0.98–1.04), 0.380
		High ALT	**1.08 (1.00–1.16), 0.049**	1.02 (0.99–1.04), 0.191
Any	**1.07 (1.00–1.14), 0.048**	High AST	1.03 (0.96–1.12), 0.390	**1.03 (1.00–1.06), 0.023**
		High ALT	1.05 (0.97–1.13), 0.220	1.02 (1.00–1.05), 0.076
Predictor: HCV infection				
Nuclear	**1.35 (1.18–1.55), <0.001**	High AST	**1.39 (1.17–1.64), <0.001**	0.97 (0.92–1.03), 0.346
		High ALT	**1.35 (1.16–1.57), <0.001**	1.00 (0.98–1.03), 0.955
Any	**1.40 (1.12–1.76), 0.003**	High AST	**1.42 (1.09–1.85), 0.010**	0.99 (0.93–1.05), 0.759
		High ALT	**1.40 (1.10–1.78), 0.006**	1.00 (0.97–1.03), 0.864

Table shows ORs (95% CIs), *P* values. ^a^Bold indicates significance. Adjusted for age, sex, smoking amount, alcohol consumption, metabolic equivalence task, education, household income, diabetes mellitus, obesity, sun exposure, and family history of eye diseases. ^b^Pathways are described in Fig. 2. ^c^High aspartate aminotransferase (AST) is defined as AST >49.9 IU/L. ^d^High alanine aminotransferase (ALT) is defined as ALT >56.1 IU/L. Goodness of fit measures for all mediation models were: the models’ relative χ^2^ = 1.34 to 1.97 with *P* values > 0.05, RMSEA = 0.006 to 0.010, and CFI = 0.992 to 0.999. The mediation analysis results for the relationship between HBV infection and any cataract was also shown in Fig. 2.

## Discussion

This study shows that both HBV and HCV infection are significantly associated with nuclear and any cataract; high AST may be a complete and important mediator of the association between HBV and any cataract. These findings imply that people infected with HBV or HCV should be examined and carefully observed for cataract formation in terms of ocular health. In line with our findings, a previous study also suggested the positive association of HCV infection and the development of age-related cataract^13^.

In the relationship between HBV infection and any cataract, high AST levels completely mediated the effects of HBV infection on any cataract. Several studies support the biological plausibility that HBV infection induces cataract formation by hepatic fibrosis and inflammation. HBV infection and HBV DNA levels develop hepatic fibrosis and inflammation, and AST levels are the best predictor for significant inflammation^14^. In addition to inflammation, oxidative stress and induced cellular damage, which are hallmarks of chronic inflammatory processes, increase the risk of age-related cataract formation^15^. In addition, endothelial dysfunction caused by oxidative stress and inflammatory processes is also likely to develop age-related cataract^16^. Sartori *et al*. proposed that active infection of HBV and HCV retain an inflammatory status developing endothelial damage^17^.

Interestingly, the relationships of HBV infection-nuclear cataract, of HCV infection-nuclear cataract, and of HCV infection-any cataract were not explained by liver damage; rather, these associations may be explained by other mechanisms. A number of studies have reported the detection of HBV and HCV antigen in tears and aqueous humor of seropositive patients^18,19^. Furthermore, HBV infection has significant associations with several ocular pathophysiology^20^ such as age-related macular degeneration^21^ and dry eye disease^22^. Compared to HBV infections, HCV infection has association with a greater variety of ocular pathophysiology^20,23^, such as acute loss of vision^24^, retinopathy^25^, retinal pigment epitheliitis^26^, uveitis^27^, and dry eye disease^28^. Although our study did not suggest mechanisms other than AST or ALT routes, we assume that there is a common pathophysiological pathway among hepatitis-induced ocular diseases. Alternatively, we could explain that the risk of age-related cataract among the HBV or HCV infected patients could associate with the use of interferon to treat hepatitis infections^20,23^. Zegans *et al*. proposed that adverse interferon-related ocular effects include vitreous and subconjunctival hemorrhage, retinal microaneurysms, panophthalmitis, and central retinal vein occlusion^23^. Indeed, one study reported cataract as a consequence of interferon therapy in HCV patients co-infected with HIV^29^. Another study observed the development of posterior subcapsular cataract in a patient with HCV infection during treatment^30^. Nevertheless, since only 0.3% (n = 32) and 0.06% (n = 2) of our study population were under treatment for HBV and HCV infection, respectively, we did not adjust the status of treatments for these infections in the statistical analyses. Instead, when we performed χ^2^ tests to evaluate the associations of treatments for HBV and HCV infection with nuclear and any cataract, results were not significant (data not shown).

It is unclear whether there are relationships between HBV and HCV infection and cataract formation and whether liver function modifies these relationships. To our knowledge, this is the first study using mediation analysis to examine these relationships. However, one limitation of this study was that the current cross-sectional study cannot prove any causal relationships. Another limitation was that we did not perform a “rare events” estimation in the mediation analysis using a penalized-likelihood approach, such as the Firth method, due to the limited development of structural equation model methodology. A final limitation was that because the increase of ORs of age-related cataract by HBV was not high, HBV-relevant interventions for cataract may be limited in preventing cataract.

Our findings showed that hepatitis virus infections and liver damage, accordingly, were highly associated with cataract formation. Furthermore, our study additionally shows that liver damage can be the crucial mediator in the relationship of hepatitis virus infections with cataract. Screening for HBV and HCV needs to be considered in order to prevent and treat people against age-related cataract. Future prospective studies need to elucidate the differences among the demonstrated relationships and the role of liver damage in hepatitis virus infection-cataract association in other populations.

## Methods

This study analyzed data in the Korea National Health and Nutrition Examination Survey (KNHANES) 2010–2012. KNHANES is a series of cross-sectional survey to investigate nationwide representative data on the health and nutritional status of the Korea population. Survey population was selected by stratified multi-stage design according to age, sex, and geographic region. This survey consists of health interviews and examinations and dietary interviews which are performed by physicians and trained research staff. Following Ethical Principles for Medical Research Involving Human Subjects defined by the Declaration of Helsinki, an individual informed consent form was obtained from all participants of KNHANES. A detailed description of the survey methods is provided elsewhere^31^. As KNHANES produces deidentified public data, our study does not require the approval of the institutional review board.

Among participants of KNHANES 2010–2012, 11,820 participants aged ≥40 years completed examinations for HBV or HCV infection and cataract. Of these, this study excluded data on 1,783 participants who had ever been diagnosed as having cataract or had experienced cataract surgery, because we assumed that this eye status may have developed before their present hepatitis or hepatic status was formed. Therefore, the final study population included 10,037 participants (4,435 men and 5,602 women). As HBV was examined in KNHANES 2010–2012 while HCV was examined only in 2012, the numbers of participants were different in investigations of HBV-cataract (n = 10,037) association and HCV-cataract (n = 3,166) association.

In KNHANES, each participant was serologically screened for HBV infection by measuring HBsAg using an electrochemiluminescence immunoassay system (E-170; Roche, Germany) and for HCV infection by measuring anti-HCV using a chemiluminescent microparticle immunoassay (ARCHITECT i4000Sr; ABBOTT, Germany). To evaluate liver function status, AST and ALT were measured by a UV method using a Hitachi Automatic Analyzer 7600 (Hitachi, Japan) and dichotomized into normal (AST ≤ 49.9 IU/L, ALT ≤ 56.1 IU/L) and high (AST > 49.9 IU/L, ALT > 56.1 IU/L) levels.

Cataract was diagnosed by using a slit-lamp microscope (Haag-Streit model BQ-900; Haag-Streit, Koeniz, Switzerland). When a participant had any subtype of cataract in either eye, he or she was defined as having cataract as explained in a previous study^32^. Subtypes of cataract were classified into cortical [Lens Opacities Classification System III (LOCS-III) score ≥4], nuclear (LOCS-III score ≥4 for nuclear opalescence or ≥4 for nuclear color), anterior subcapsular (LOCS-III score ≥0.6), posterior subcapsular (LOCS-III score ≥2), and mixed (≥2 subtypes) compared with photographic standards. “Any” cataract was defined as having any of the 5 subtypes in either eye.

**Figure 3:**
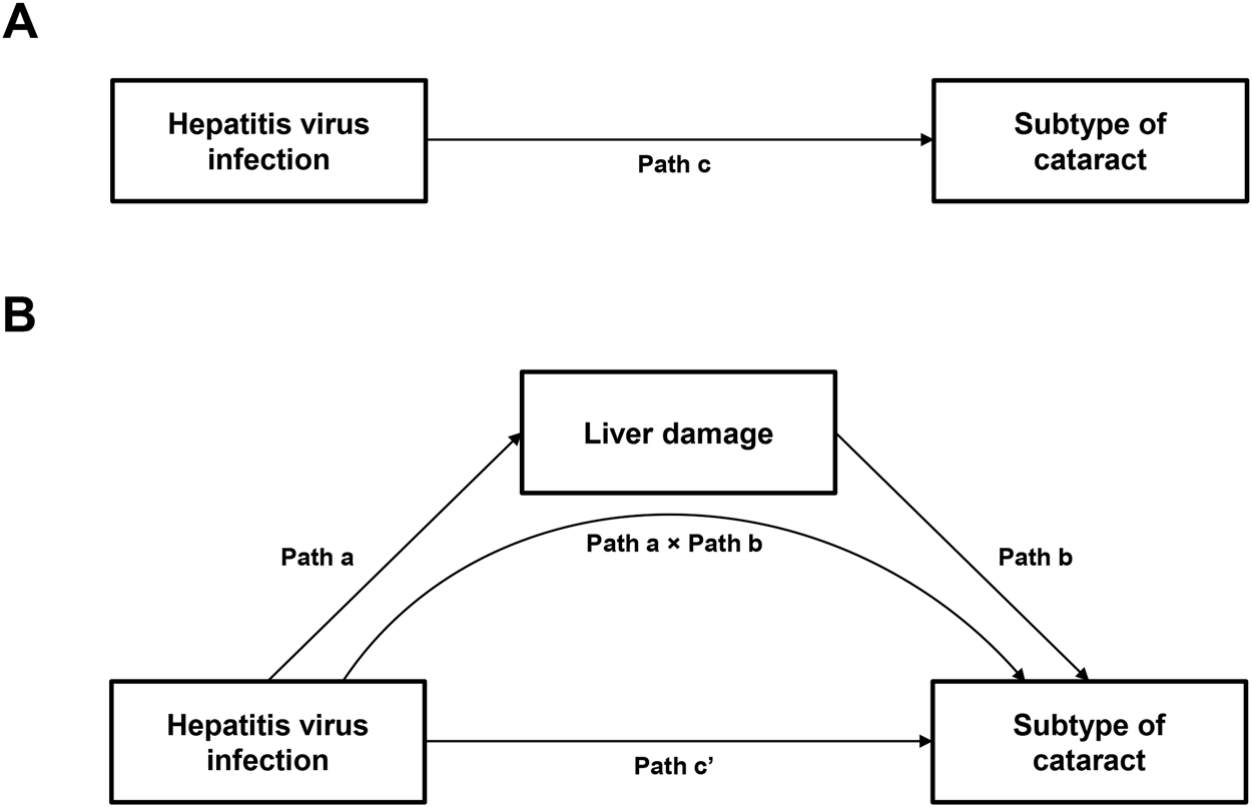
Hypothesized mediation pathway in the association between hepatitis B virus (HBV) or hepatitis C virus (HCV) infection and subtype of cataract through liver damage. (**A**) Total effect (Path c) of HBV or HCV infection on cataract, (**B**) Direct (Path c’) and indirect (Path a × Path b) effects of HBV or HCV infection on cataract through liver damage measured by high aspartate aminotransferase (AST, >49.9 IU/L) or high alanine aminotransferase (ALT, >56.1 IU/L).

We compared cataract prevalence between participants infected and uninfected by HBV or HCV after adjusting for age, sex, smoking amount, alcohol consumption, metabolic equivalence task, education, household income, diabetes mellitus, obesity, sun exposure, and family history of eye diseases using multivariable logistic regressions. However, we did not performed statistical analysis for anterior and posterior subcapsular cataracts because of the low prevalence [0.94% (n = 94) and 0.45% (n = 45), respectively]. Mediation analysis was performed only when the total effect of HBV or HCV on a subtype of cataract was significant [Fig. 3(A)]. In mediation analysis, we evaluated whether and to what extent the relationship between HBV or HCV infection and subtype of cataract is explained by an indirect effect, such as high AST and ALT [Fig. 3(B)]. We defined “complete mediation” as a significance of indirect effect, indicated as Path a × Path b in Fig. 3(B), tested by Sobel test and a lack of significance in the association between HBV or HCV and a subtype of cataract^33^. We defined “partial mediation” as a significance of indirect effect tested by Sobel test and a significance in the association between HBV or HCV and a subtype of cataract^33^. Mediation analysis using structural equation models was performed with the same adjustments for the covariates as above. To estimate the parameters of the model, we used maximum likelihood (ML) weighted least square with means and variance adjusted estimation (WLSMV). The following indicators are recommended to test the goodness-of-fit of the model: the model’s relative χ^2^ (χ^2^/df) (satisfactory, 3:1) with *P* value > 0.05, RMSEA (satisfactory, <0.07), and CFI (satisfactory, >0.95)^34^. All statistical analyses were performed using SAS software, version 9.4 (SAS Institute; Cary, NC), with exception of the mediation analysis, which was performed by using Mplus 7.3 statistical software (Muthén and Muthén 1998–2014). A *P* value < 0.05 was considered to be statistically significant.
